# Fiber modifications enable fowl adenovirus 4 vectors to transduce human cells

**DOI:** 10.1002/jgm.3368

**Published:** 2021-06-11

**Authors:** Wenfeng Zhang, Xiaojuan Guo, Fengcai Yin, Xiaohui Zou, Wenzhe Hou, Zhuozhuang Lu

**Affiliations:** ^1^ School of Laboratory Medicine Weifang Medical University Weifang China; ^2^ State Key Laboratory of Infectious Disease Prevention and Control National Institute for Viral Disease Control and Prevention, Chinese Center for Disease Control and Prevention Beijing China; ^3^ Henan Chemical Technician College Kaifeng China; ^4^ Chinese Center for Disease Control and Prevention‐Wuhan Institute of Virology Chinese Academy of Sciences Joint Research Center for Emerging Infectious Diseases and Biosafety Wuhan China

**Keywords:** fiber, fowl adenovirus 4, gene transduction, genetic modification, vector, virus binding

## Abstract

**Background:**

Pre‐existing immunities hamper the application of human adenovirus (HAdV) vectors in gene therapy or vaccine development. Fowl adenovirus (FAdV)‐based vector might represent an alternative.

**Methods:**

An intermediate plasmid containing FAdV‐4 fiber genes, pMD‐FAV4Fs, was separated from FAdV‐4 adenoviral plasmid pKFAV4GFP. An overlap extension polymerase chain reaction (PCR) was employed for fiber modification in pMD‐FAV4Fs, and the modified fibers were restored to generate new adenoviral plasmids through restriction‐assembly. FAdV‐4 vectors were rescued and amplified in chicken LMH cells. Fluorescence microscopy and flow cytometry were used to evaluate the gene transfer efficiency. The amount of viruses binding to cells was determined by a real‐time PCR. A plaque‐forming assay and one‐step growth curve were used to evaluate virus growth.

**Results:**

Four sites in the CD‐, DE‐, HI‐ and IJ‐loop of fiber1 knob could tolerate the insertion of exogenous peptide. The insertion of RGD4C peptide in the fiber1 knob significantly promoted FAdV‐4 transduction to human adherent cells such as 293, A549 and HEp‐2, and the insertion to the IJ‐loop demonstrated the best performance. The replacement of the fiber2 knob of FAdV‐4 with that of HAdV‐35 improved the gene transfer to human suspension cells such as Jurkat, K562 and U937. Fiber‐modified FAdV‐4 vectors could transduce approximately 80% human cells at an acceptable multiplicity of infection. Enhanced gene transfer mainly resulted from increased virus binding. Fiber modifications did not significantly influence the growth of recombinant FAdV‐4 in packaging cells.

**Conclusions:**

As a proof of principle, it was feasible to enhance gene transduction of FAdV‐4 vectors to human cells by modifying the fibers.

## Graphical abstract

Fowl adenovirus 4 (FAdV‐4) vectors can hardly transduce human cells. Incorporation of RGD4C peptide to the IJ loop of fiber1 enabled FAdV‐4 to transduce human adherent cells, and the replacement of fiber2 knob with that of HAdV‐35 enhanced the transduction of FAdV‐4 to human suspension cells.

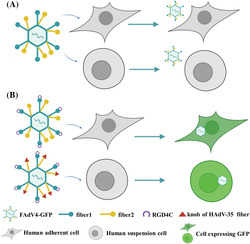

## 1 INTRODUCTION

Adenoviruses are non‐enveloped viruses containing linear double‐stranded DNA genomes of 26–48 kb.^1^ The family *Adenoviridae* consists of five genera, among which Mastadenoviruses infect mammalian hosts exclusively, whereas Aviadenoviruses have been found only in birds.^2^ Human adenoviruses (HAdVs) have been comprehensively studied and are constructed as gene transfer vectors for gene therapy and vaccine development.^3^, ^4^, ^5^ Adenoviral vectors have many advantages compared to other viral gene transfer tools: the medium‐sized genomes are manipulable and suitable for stably loading transgene up to 7 kb or even longer; they can be produced with a high yield and reasonable cost; and their non‐enveloped virions, with physicochemical stability, are convenient for preservation and usage. Moreover, adenoviruses feature efficient gene transfer to cells at a high multiplicity of infection (MOI) and adenoviral vectors do not integrate into chromosomes, which reduces the risk of causing cellular transformation. However, the pre‐existing immunity against HAdV impairs the efficacy and safety of these vectors in clinical practice.^6^, ^7^

HAdVs are ubiquitous pathogens and generally cause mild, self‐limiting infections in immunocompetent people.^8^, ^9^ HAdV‐5‐based vectors have been commonly used. The seroprevalence of HAdV‐5 neutralizing antibody (NAb) varies in different geographic regions and was reported to be 50–90% in healthy adults.^10^, ^11^, ^12^ Such a situation has attracted interest regarding the development of vectors based on HAdV serotypes with a lower seroprevalence or on chimpanzee adenoviruses (ChAdVs).^13^, ^14^, ^15^, ^16^ Recent epidemiological data demonstrate that geographical distributions of pathogens are related to climate, temporal variation and immigration and are not limited by geopolitical borders. For example, HAdV‐D26 is generally considered to have low seroprevalence and HAdV‐D26 NAb was detected to be positive in less than 12% of the sera collected from the USA and European countries. However, the HAdV‐D26 seroprevalence was 70–90% in some African countries and 35–60% in China and Thailand.^11^ Even though non‐human primate (NHP) adenoviruses are less likely to infect human, the pre‐existing immunity in human was detected as a result of the cross‐reactivity between HAdV and ChAdV.^11^, ^17^ Therefore, we need to address the pre‐existing immunity for clinical application of both HAdV and NHP adenovirus vectors.

Fowl adenoviruses (FAdVs) have the potential to be constructed as vectors targeting human cells, whereas these vectors have low gene transfer efficiency. FAdVs belong to the genus of Aviadenovirus^1^ and 12 serotypes (FAdV‐1 to ‐8a and ‐8b to 11) of FAdV have been identified, which belong to five species (FAdV‐A to ‐E).^18^ CELO (chicken embryo lethal orphan) virus is the model type of FAdV‐A1 and has been constructed as gene transfer vectors.^19^ FAdV vectors have two advantages over HAdV counterparts: a lack of pre‐existing immunity against FAdV in human and a low cost of producing FAdV in chicken embryo, although the biosafety of FAdVs has not been studied. It was reported that FAdV‐1 could infect mammalian cells by binding to CAR receptors with its fiber1, although doubts were raised by the results of crystal structure studies.^20^, ^21^ FAdV‐1 vectors, even with a modified fiber1, could only transduce mammalian cells with limited efficiency.^22^

Fiber modification can change the tropism of adenoviruses because fiber is the main ligand for virus to bind to its cellular receptor. Arginine‐glycine‐aspartic acid (RGD) peptide family is known as the most prominent ligand for the extracellular domain of integrin receptors.^23^, ^24^, ^25^, ^26^, ^27^ RGD4C peptide can bind to integrin with high affinity and promote adenovirus infection when incorporated into the fiber knob.^4^, ^28^, ^29^ However, the incorporation of RGD4C into the receptor binding site of the fiber knob (e.g. the insertion of RGD4C into the HI loop of HAdV‐35 fiber knob) would impair the interaction between fiber and its native receptor.^30^

We established a vector system based on FAdV‐4, and found that fiber1 was the essential gene, whereas fiber2 was dispensable for FAdV‐4 infection.^31^ Because FAdV‐4 could hardly transduce human cells, we attempt to improve the transduction of FAdV‐4 vector to adherent or suspension human cells by incorporation of RGD4C oligopeptide to fibers or replacement of fiber knob.

## 2 MATERIALS AND METHODS

### 2.1 Plasmids, oligonucleotides and reagents

pKFAV4GFP, pKFAV4‐CX19A, pMD‐FAV4Fs and pFiber5‐35 were constructed previously. pKFAV4GFP was an adenoviral plasmid carrying FAdV‐4 genome, in which the sequence of FAdV‐4 ORF1, ORF1B and ORF2 was replaced with cytomegalovirus (CMV) promoter‐controlled green fluorescent protein (GFP) expression cassette. ORF19A coding sequence (CDS) was deleted and GFP CDS was replaced with that of mCherry in pKFAV4GFP to generate pKFAV4‐CX19A.^32^ pMD‐FAV4Fs, a shuttle plasmid for FAdV‐4 fiber modification, carried MauBI‐SbfI fragment and short sequences flanking this region in the FAdV‐4 genome.^31^ pFiber5‐35 carried the sequence encoding the fiber knob of HAdV‐35 and was used as the template for polymerase chain reaction (PCR) cloning of HAdV‐35 fiber knob.^33^

Single‐stranded DNA oligonucleotides were designed, synthesized and used as PCR primers (Table 1). PCR was routinely performed for gene cloning (Q5 High‐Fidelity DNA Polymerase, Cat. M0491S; New England Biolabs, Ipswich, MA, USA) or plasmid identification before sequencing (Premix Taq, Cat. RR901A; TaKaRa, Dalian, China). Plasmid construction was performed with Gibson assembly (NEBuilder HiFi DNA Assembly Master Mix, Cat. E2621; New England Biolabs) or restriction‐ligation cloning (DNA Ligation Kit, Cat. 6022Q; TaKaRa). DNA recovery and cleaning were performed with kits from Zymo Research (Cat. D4045 and D4010; Zymo Research, Irvine, CA, USA). Restriction enzymes were purchased from New England Biolabs or TaKaRa. Plasmid transformation into *Escherichia coli* TOP10 chemically competent cells was conducted in accordance with the heat shock procedure described in the manufacturer's instructions (TIANGEN Biotech, Beijing, China).

**TABLE 1:** Summary information of PCR primers

Fragment	Primer name	Sequence	Template	Product (bp)	Annotation
SacI‐CDR	1805FAV4F1Mf	gtccataggc tattacatct acatggtg	pMD‐FAV4Fs	119	*Sac*I
	1805FAV4F1CDRr	agaaacagtc tccgcggcag tcacagctcg cgcctgtgag gtc			
CDR‐AgeI	1805FAV4F1CDRf	tgactgccgc ggagactgtt tctgcggaga aaacagcctg actagcg	pMD‐FAV4Fs	475	
	1805FAV4F1Mr	accctgatag gaaaaaggga tagga			*Age*I
SacI‐DER	1805FAV4F1Mf	gtccataggc tattacatct acatggtg	pMD‐FAV4Fs	209	*Sac*I
	1805FAV4F1DERr	agaaacagtc tccgcggcag tcacagatga gggacaaatt cacctctgt			
DER‐AgeI	1805FAV4F1DERf	tgactgccgc ggagactgtt tctgcgtgcc gcccacggtc tc	pMD‐FAV4Fs	385	
	1805FAV4F1Mr	accctgatag gaaaaaggga tagga			*Age*I
SacI‐HIR	1805FAV4F1Mf	gtccataggc tattacatct acatggtg	pMD‐FAV4Fs	443	*Sac*I
	1805FAV4F1HIRr	agaaacagtc tccgcggcag tcacatccgc tggacgtgtt ctgg			
HIR‐AgeI	1805FAV4F1HIRf	tgactgccgc ggagactgtt tctgcaccac gccgtcggat gc	pMD‐FAV4Fs	151	
	1805FAV4F1Mr	accctgatag gaaaaaggga tagga			*Age*I
SacI‐IJR	1805FAV4F1Mf	gtccataggc tattacatct acatggtg	pMD‐FAV4Fs	521	*Sac*I
	1805FAV4F1IJRr	agaaacagtc tccgcggcag tcacacgcgt tctggtcaaa ccagtt			
IJR‐AgeI	1805FAV4F1IJRf	tgactgccgc ggagactgtt tctgccccga cactgtggtg acgac	pMD‐FAV4Fs	73	
	1805FAV4F1Mr	accctgatag gaaaaaggga tagga			*Age*I
MauBI‐F2CDR	1811MD‐FAV4FSf	ccggtacctt tcggagggcg acg	pMD‐FAV4Fs	2245	*Mau*BI
	1811MD‐F2CDRp2	cagaaacagt ctccgcggca gtcacaccca gggcgattcc ccatgg			
F2CDR‐SbfI	1811MD‐F2CDRp3	gtgactgccg cggagactgt ttctgcgacc tcaactccgc caatgccaaa t	pMD‐FAV4Fs	787	
F1‐F2CDR	1811MD‐FAV4FSr	gatatcctcc tttggaccca tggtagtcca c		3007	*Sbf*I
F1IJR‐F2TS	1811MD‐FAV4FSf	ccggtacctt tcggagggcg acg	pKFAV4F1IJR‐GFP	2084	*Mau*BI
	1905F2TSF35Kp2	ggtgttaata ctatcgggtg tggagacgct cccc			
F35K	1905F2TSF35Kp3	gcgtctccac acccgatagt attaacacct tatggactgg	pFiber5‐35	617	
	1905F2TSF35Kp4	gatcatggtt ccgctttagt tgtcgtcttc tgtaatgtaa g			
F2‐SbfI	1905F2TSF35Kp5	aagacgacaa ctaaagcgga accatgatcg tgg	pMD‐FAV4Fs	418	
F1IJR‐F35K	1811MD‐FAV4FSr	gatatcctcc tttggaccca tggtagtcca c		3061	*Sbf*I
Fiber‐seq	1805FAV4FMS1	taaccaatat cttctaggct ccg	plasmids or viruses with RGD4C insertion	1436	For identification
	1805FAV4FMS2	gaaaggataa accaggtcaa gc		or 1463	
GFP‐frag	2008GFPf	gacaaccact acctgagcac cc	virus genomic DNA	126	
	2008GFPr	cttgtacagc tcgtccatgc c			
	2008GFP‐probe	tccgccctga gcaaagaccc caac			5′HEX, 3′BHQ1

### 2.2 Cell culture

Adherent cells, including 293 (CRL‐2117; ATCC, Manassas, VA, USA), A549 (CCL‐185), HEp‐2 (ATCC CCL‐23) and LMH (CRL‐2117) were cultured in Dulbecco's modified Eagle's medium (DMEM) plus 10% fetal bovine serum (FBS) (HyClone, Logan, UT, USA) at 37°C in a humidified atmosphere supplemented with 5% CO_2_. Suspension cells, including HL‐60 (acute myelogenous leukemia), Jurkat (T‐cell leukemia), K562 (chronic myelogenous leukemia) and U937 (promonocytic leukemia) were cultured in a similar manner, except that DMEM was replaced with RPMI 1640. Cells were split twice a week. To help the LMH cells attach, flasks or plates were precoated with 0.1% gelatin (Cat. G9391; Sigma‐Aldrich, St Louis, MO, USA). Adherent cells were split at a ratio of 1:2.5 the day before transfection or viral infection. On reaching 80% confluency, the cells were transfected with linearized adenoviral plasmid mixed with jetPRIME reagent in accordance with the manufacturer's instructions (Cat. 114‐15; Polyplus‐transfection, Illkirch, France) or infected with recombinant virus. The suspension cells in the logarithmic growth phase were counted and used for viral infection.

### 2.3 Plasmid construction

We took the insertion of RGD4C CDS to fiber1 CD loop as an example to determine the procedure of fiber1 modification (see Supporting information, Figure S1). With pMD‐FAV4Fs as the template, PCR was performed using the primer 1805FAV4F1Mf/1805FAV4F1CDRr to amplify SacI‐CDR fragment (119 bp) and the primer 1805FAV4F1CDRr/1805FAV4F1Mr to amplify CDR‐AgeI fragment (475 bp). pMD‐FAV4Fs was digested with *Sac*I/*Age*I, and then the fragment of 5124 bp was recovered after electrophoresis, mixed with the SacI‐CDR and CDR‐AgeI fragments and subjected into DNA assembly to generate the modified shuttle plasmid pMD‐FAV4F1CDR. pMD‐FAV4F1CDR was digested with *Kpn*I/*Eco*RV to recover fragment containing fibers (2997 bp), pKFAV4GFP was digested with *Mau*BI/*Sbf*I to recover the large fragment (43,078 bp) and these two fragments were mixed for DNA assembly to generate adenoviral plasmid pKFAV4F1CDR‐GFP (restriction‐assembly; see Supporting information, Figure S2).^34^, ^35^ To insert RGD4C into the fiber2 CD loop, overlap extension PCR was performed to amplify F1‐F2CDR fragment (3007 bp), which was inserted at the *Mau*BI/*Sbf*I sites in pKFAV4GFP to generate pKFAV4F2CDR‐GFP through DNA assembly (see Supporting information, Figure S3). The intermediate plasmid‐based system was employed for replacing the CMV promoter with human EF1a promoter to generate adenoviral plasmid pKFAV4‐EG.^32^ pKFAV4‐EG was a progeny plasmid of pKFAV4‐CX19A and different from pKFAV4‐GFP in ORF0 and ORF19A deletion and the replacement of GFP promoter (the detailed procedure will be provided upon request). F1IJR‐F2 fragment was amplified by PCR with pKFAV4F1IJR‐GFP as the template and primers 1811MD‐FAV4FSf/r and inserted at the *Mau*BI/*Sbf*I sites in pKFAV4‐EG to generate pKFAV4F1IJR‐EG by DNA assembly. The F1IJR‐F35K fragment was amplified by overlap extension PCR (Table 1) and inserted at the *Mau*BI/*Sbf*I sites in pKFAV4‐EG to generate pKFAV4FIJ35K‐EG by DNA assembly.

### 2.4 Rescue, purification and titration of recombinant viruses

Adenoviral plasmid was linearized by *Pme*I digestion, recovered and used to transfect LMH cells. GFP focuses could be observed under fluorescence microscope 3 days post transfection. The cells together with culture medium were harvested 2–4 days later, subjected to three freeze–thaw cycles and centrifuged to remove cellular debris. The seed virus was amplified in LMH cells, purified with the traditional ultracentrifugation method except that 10 mm citrate (pH 6.2) instead of 10 mm Tris‐Cl (pH 7.6) was used as the buffer solution.^32^ Particle titer of purified virus was determined by measuring the content of genomic DNA, where 100 ng of genomic DNA is equivalent to 2.3 × 10^9^ viral particles (vp) because a 43‐kb genome has a molecular mass of 2.6 × 10^7^. Infectivity titer was determined using LMH cells with the limiting dilution assay by counting GFP+ cells 30 hours post infection.^36^

### 2.5 Transduction experiments

Adherent cells were seeded on 24‐well plates and infected with recombinant virus at the indicated MOIs (vp/cell) in 0.25 ml of DMEM plus 2% FBS. The culture was stirred once every 1 hour to facilitate virus diffusion. The virus was removed and fresh DMEM containing 2% FBS in 0.5 ml was added to each well 4 hours post infection. The expression of reporter gene was observed and photographed under a fluorescence microscope, 48 hours post infection. The cells were washed once with 10 mm phosphate‐buffered saline (PBS), detached with 0.25% trypsin in 0.3 ml of PBS, and dispersed into single cell suspension by aspiration, to which 0.2 ml of PBS containing 2.5% FBS was added to prevent cell reaggregation, and 0.3 ml of 4% paraformaldehyde in PBS was further added to fix the cells. The expression of GFP was analyzed by flow cytometry within 1 week. For suspension cells, 2.5 × 10^5^ cells were infected with virus at the indicated MOIs in 0.25 ml RPMI 1640 plus 2% FBS, stirred once every 1 hour for virus diffusion in the following 4 hours and supplemented with 0.25 ml of fresh medium containing 2% FBS without removal of the virus 24 hours post infection. The GFP expression was similarly determined, except that trypsin treatment was spared.

### 2.6 Virus binding assay

The cultured cells were cooled down at 4°C for 1 hour and then mixed with precooled virus diluents (virus in culture medium containing 2% FBS) as described above. The virus binding assay was carried out with agitation on a rocker at 4°C for 2 hours. The virus‐containing medium was removed by aspiration (for adherent cells) or centrifugation (for suspension cells) and the cells were washed twice with precooled PBS. Virus genomic DNA was extracted from cells (QIAamp MinElute Virus Spin Kit, Cat. 57704; Qiagen, Germantown, MD, US) and used as the template for real‐time PCR (Probe qPCR Mix, Cat. RR391A; TaKaRa), which was performed with the primers and probe binding to GFP gene (2008GFPf/r, 2008GFP‐probe) (Table 1) to determine the copy number of viral genome. The copy number of input viruses was calculated by multiplying the diluted particle titer by the incubation volume, and the virus binding ratio was defined as the copy number of adsorbed viruses divided by that of input ones.

### 2.7 Plaque‐forming experiment

The LMH cells in six‐well plates were covered with 1 ml of DMEM containing 2% FBS and recombinant virus of 20,000 or 40,000 vp on a rocker at room temperature for 2 hours. The virus diluent was discarded, and the cells were washed twice with PBS and then covered with 2 ml of DMEM containing 2% FBS and 1% low‐melting agarose (SeaPlaque agarose, Cat. 50100; BioWhittaker Molecular Applications, Rockland, ME, US). After 4 days, 2 ml of fresh liquid DMEM plus 2% FBS was supplemented to each well without disturbance of the lower semi‐solid layer. After 7 days, liquid culture medium was removed carefully and 2.5 ml of 4% paraformaldehyde in PBS was added to each well to fix the cells. The cells were then stained with crystal violet solution,^37^ and the plaques were photographed. The areas of the plaques were measured by using Fiji image processing package (http://fiji.sc) and compared by the Kruskal–Wallis nonparametric test.

### 2.8 Statistical analysis

The data are presented as the mean ± SD unless otherwise indicated. The data collected from one cell line were combined and analyzed with two‐way analysis of variance. MOI and virus type were defined as two factors, and Dunnett's multiple comparisons test was performed to compare the difference of mean values between virus types at each MOI. *p* < 0.05 was considered statistically significant.

## 3 RESULTS

### 3.1 Construction of adenoviral plasmids

**FIGURE 1:**
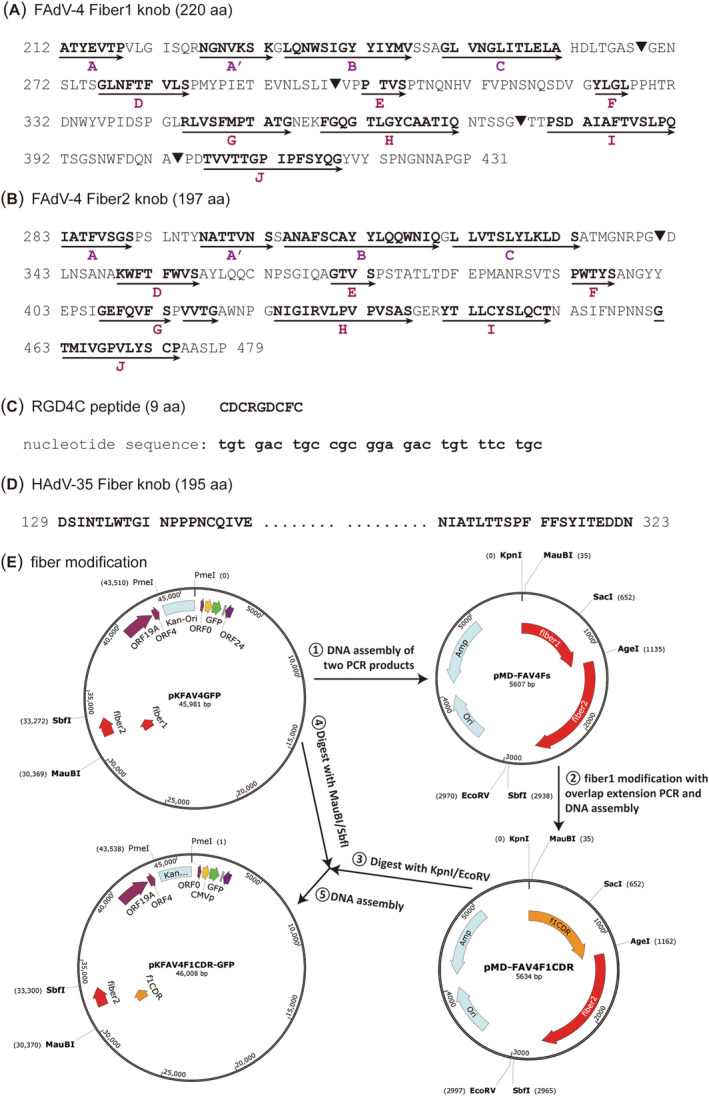
Schematic diagram of fiber modification sites, elements and procedure. (A) Amino acid sequence of the FAdV‐4 fiber1 knob. The β‐strands, which are shown as bold letters and denoted with arrows, were predicted according to the alignment of FAdV fiber knob domains and the crystal structures of FAdV‐1 fiber1 (PDB ID 2IUN) and fiber2 (PDB ID 2VTW).^38^ The sites for RGD4C insertion are labelled with a solid inverted triangle “▼”. (B) Amino acid sequence of FAdV‐4 fiber2 knob. The site for RGD4C insertion is labelled with a solid inverted triangle “▼”; and the whole knob is replaced with that of HAdV‐35 fiber in another construction. (C) Amino acid and coding sequence of RGD4C peptide. (D) Amino acid sequence of HAdV‐35 fiber knob. The sequence in the middle is abbreviated; for full annotation, please refer to the GenBank sequence of AC_000019. (E) Procedure for modification of FAdV‐4 fiber1 knob. The genome of FAdV‐4 in pKFAV4GFP originated from FAdV‐4 isolate NIVD2 (GenBank MG547384)^32^

The fiber knob domains of FAdV‐1, −4 and −9 have been aligned.^38^ After checking the spatial position of every amino acid according to the crystal structures of FAdV‐1 fiber1 (PDB ID 2IUN) and fiber2 (PDB ID 2VTW),^20^, ^21^ we chose some sites in CD, DE, HI and IJ loops of the fiber1 knob for incorporation of RGD4C peptide (Figure 1A). To determine whether fiber2 knob could be similarly modified, we selected the CD loop in fiber2 for RGD4C insertion (Figure 1B and C). To increase the transduction efficiency of FAdV‐4 to human suspension cells, we replaced the whole knob of fiber2 with that of HAdV‐35 (Figure 1D). Two unique restriction sites of *Mau*BI and *Sbf*I outside the fiber region facilitated the modification. With the help of intermediate plasmid pMD‐FAV4Fs,^31^ fiber1‐modified adenoviral plasmids were constructed (Figure 1E; see also Supporting information, Figure S1 and S2). Fiber2 or fiber1/fiber2 combined modifications were carried out by DNA assembly of *Mau*BI/*Sbf*I‐digested adenoviral plasmid and the product of overlap extension PCR. In the adenoviral plasmids pKFAV4F1IJR‐EG and pKFAV4FIJ35K‐EG, the promoter of transgene (CMV promoter) was replaced with that of human EF1a gene to increase transgene expression in human suspension cells (Table 2).

**TABLE 2:** Summary information of purified FAdV‐4 vectors

Virus name	Short name	Fiber modification	Deletion of FAdV‐4 genes	Promoter of transgene	Physical titer (× 10^11^ vp/ml)	Infectivity titer (× 10^9^ IU/ml)	Particle‐to‐IU ratio
FAdV4‐GFP		None	ORF1, ORF1B, ORF2	CMV promoter	15	3.5	410
FAdV4F1CDR‐GFP	F1CDR	RGD4C in fiber1 CD loop	ORF1, ORF1B, ORF2	CMV promoter	12	2.5	480
FAdV4F1DER‐GFP	F1DER	RGD4C in fiber1 DE loop	ORF1, ORF1B, ORF2	CMV promoter	22	1.1	1900
FAdV4F1HIR‐GFP	F1HIR	RGD4C in fiber1 HI loop	ORF1, ORF1B, ORF2	CMV promoter	9.7	2.9	330
FAdV4F1IJR‐GFP	F1IJR	RGD4C in fiber1 IR loop	ORF1, ORF1B, ORF2	CMV promoter	6.6	1.3	520
FAdV4F2CDR‐GFP	F2CDR	RGD4C in fiber2 CD loop	ORF1, ORF1B, ORF2	CMV promoter	3.2	0.4	800
FAdV4F1IJR‐EG	F1IJR‐EG	RGD4C in fiber1 IR loop	ORF0, ORF1, ORF1B, ORF2, ORF19A	human EF1a promoter	15	2.5	620
FAdV4FIJ35K‐EG	FIJ35K‐EG	RGD4C in fiber1 IR loop; replacement of fiber2 knob with that of HAdV‐35	ORF0, ORF1, ORF1B, ORF2, ORF19A	human EF1a promoter	20	1.1	1900

Vp, viral particle; IU, infectious unit.

### 3.2 Preparation of recombinant FAdV‐4 viruses

**FIGURE 2:**
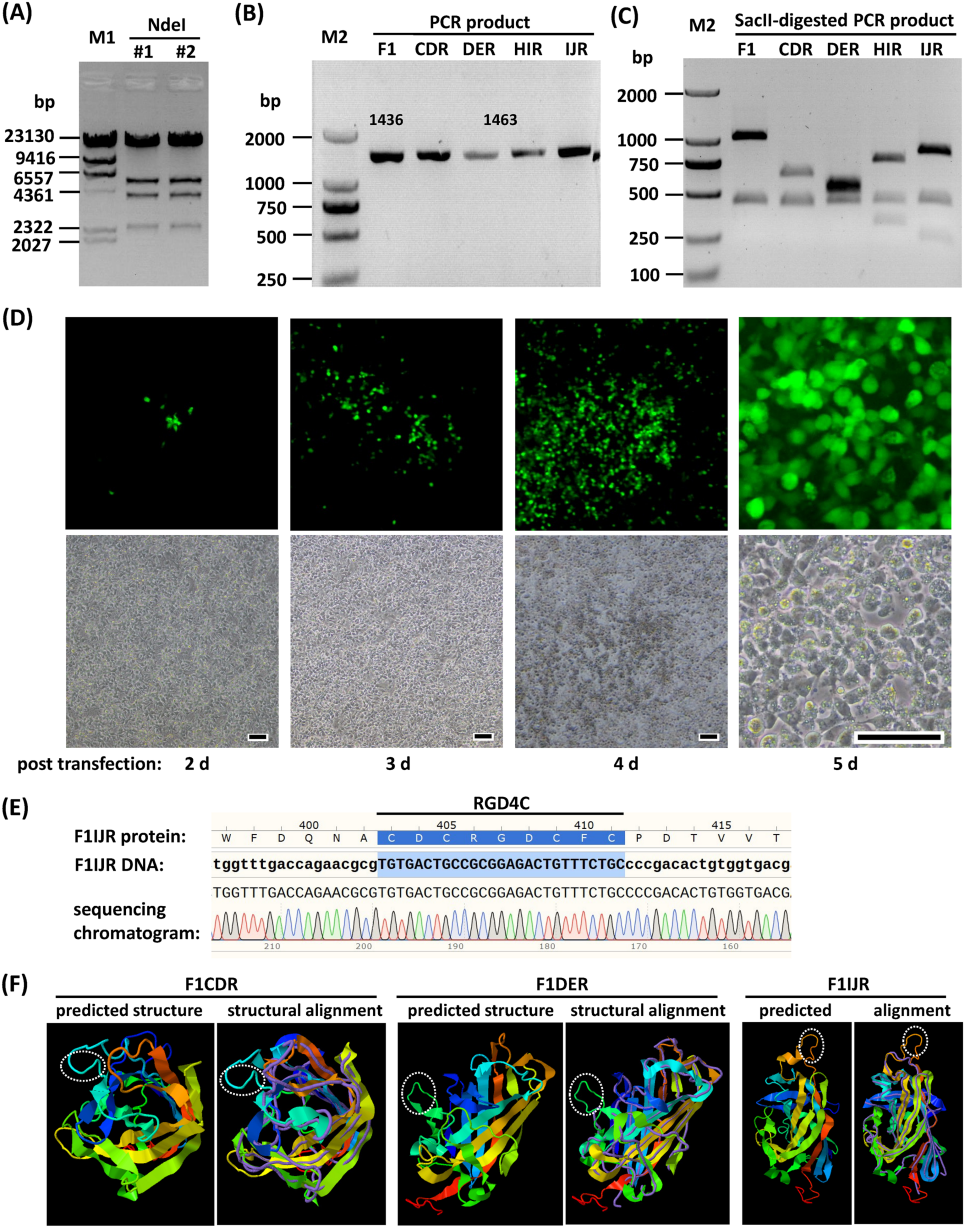
Rescue and identification of fiber‐modified FAdV‐4 vectors. (A) Restriction analysis of adenoviral plasmid pKFAV4F1CDR‐GFP. Plasmids extracted from two bacterial colonies were digested with *Nde*I and resolved on agarose gel. The expected molecular weights (bp) of the generated bands were 33,944, 5468, 3993, 2352 and 251. (B) PCR amplification of fiber region with adenoviral plasmids as the template and the primers 1805FAV4FMS1/2 (Table 1). The PCR product was 1436 bp for pKFAV4GFP (F1) or 1463 bp for fiber1 modified adenoviral plasmids (CDR, DER, HIR and IJR are short for pKFAV4F1CDR‐GFP and so on). (C) Digestion of above‐mentioned PCR products with restriction enzyme *Sac*II. The expected molecular lengths (bp) of the generated bands were 1007 and 429 for F1; 622, 429 and 412 for CDR; 532, 502 and 429 for DER; 736, 429 and 298 for HIR; and 814, 429 and 220 for IJR. (D) Rescue of FAdV4F1IJR‐GFP (F1IJR) virus in *Pme*I‐linearized pKFAV4F1IJR‐GFP‐transfected LMH cells. Scale bar = 50 μm. (E) Identification of rescued viruses by sequencing the PCR products of fiber region. A section of the sequencing chromatogram for F1IJR is shown. M1, lambda/HindIII DNA marker; M2, DL2000 DNA marker. (F) Predicted structure of RGD4C‐modified fiber. The amino acid sequences of RGD4C‐modified fiber knob were submitted to the I‐TASSER server for protein structure prediction. The reported model 1 and model‐analog structural alignment for F1CDR, F1DER and F1IJR are shown as representatives. For the structural alignments, query structure is shown in cartoon form, whereas the structural analog (PDB ID 2IUN) is displayed using backbone trace. The RGD4C motifs are marked with dotted ellipses

After identification with restriction analysis and sequencing of fiber‐modified region (Figure 2A, B and C), adenoviral plasmids were linearized and transfected into LMH cells. The growth of GFP foci could be observed under a fluorescence microscope. Enlarged GFP foci formed plaques, and a cytopathic effect occurred as plaques merged (Figure 2D). Rescued viruses were amplified, purified and titrated. The fiber modification was further confirmed by sequencing the PCR product of the fiber region in the viral genome (Figure 2E). The structure of the RGD4C‐modified fiber knob was predicted with I‐TASSER,^39^ and the results showed that the RGD4C motif protruded from the original loop, indicating an appropriate selection of insertion sites (Figure 2F). The information for purified recombinant viruses is summarized in Table 2. For the sake of convenience, short virus names are used in the present study (Table 2).

### 3.3 Transduction of adherent cells with RGD4C‐incorporated FAdV‐4 viruses

**FIGURE 3:**
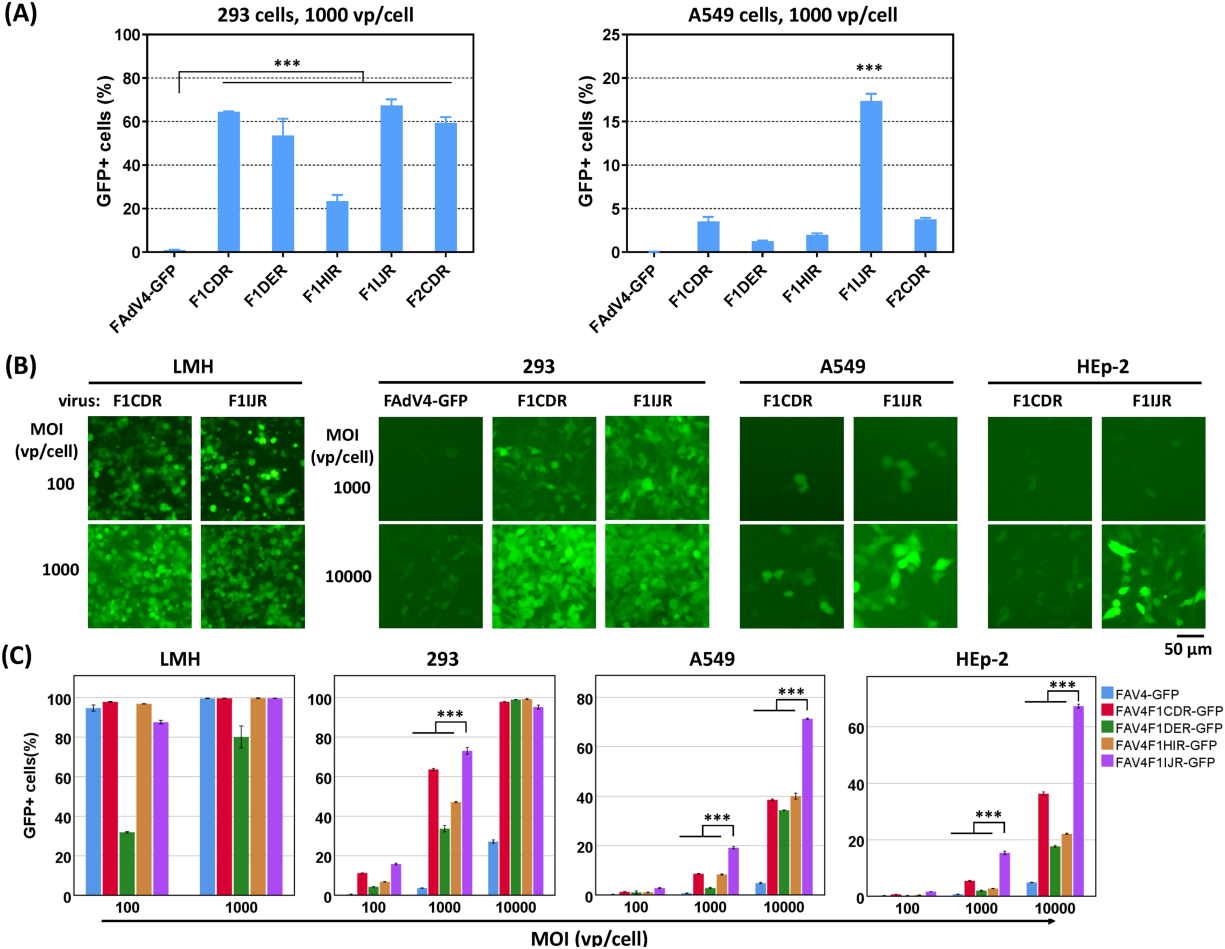
Transduction of human adherent cell lines with fiber‐modified FAdV‐4 vectors. (A) Preliminary experiments were performed with human 293 cells or A549 cells. Cells were infected with fiber modified viruses or FAdV4‐GFP (control) at a MOI (multiplicity of infection) of 1000 vp/cell (viral particle per cell) for 4 hours. Cells were detached for the preparation of single cell suspensions, and the expression of GFP was analyzed with flow cytometry 48 hours post infection. (B) Human 293, A549 and HEp‐2 cells were infected with fiber1‐modified viruses or FAdV4‐GFP at various MOIs for 4 hours. GFP expression was observed under a fluorescence microscope 48 hours post infection, and the results of F1CDR and F1IJR are demonstrated. (C) The percentages of GFP+ cells were determined by flow cytometry after microscopic observation. Chicken LMH served as the cell line control. All of the experiments were performed in duplicate and the data shown are from one representative experiment out of the three performed. ***p < 0.001

Preliminary experiments were performed with 293 and A549 cells to evaluate the gene transduction efficiency of RGD4C‐incorporated FAdV‐4 viruses (Figure 3A). FAdV‐4 could hardly transduce human cells without fiber modification (FAdV4‐GFP), and RGD4C incorporation significantly enhanced transgene delivery. F1CDR and F2CDR were equally effective for gene transduction and both were inferior to F1IJR when tested on 293 or A549 cells. Because fiber2 was planned to be reserved for knob substitution, F2CDR was excluded from further investigation. For other vectors, gene transfer efficiencies were compared on LMH, 293, A549 and HEp‐2 cells at various MOIs (Figure 3B and C). F1DER infected LMH cells with the lowest efficiency. At a MOI of 100 vp/cell, F1DER infected 30% of cells, whereas others infected 90%. F1DER was also inferior to other RGD4C‐incorporated viruses in the transduction of human cells. FAdV4‐GFP could hardly transduce human cells. At a high MOI of 10,000, FAdV4‐GFP transduced only 26% of 293 cells, 6% of A549 or 6% of HEp‐2 cells. F1IJR was superior to other viruses for all tested human cell lines. The transduction of F1IJR into A549 or HEp‐2 cells reached 70% or 65% at a MOI of 10,000, respectively.

### 3.4 Promoter and fiber2 knob substitution enhanced gene transfer to suspension cells

**FIGURE 4:**
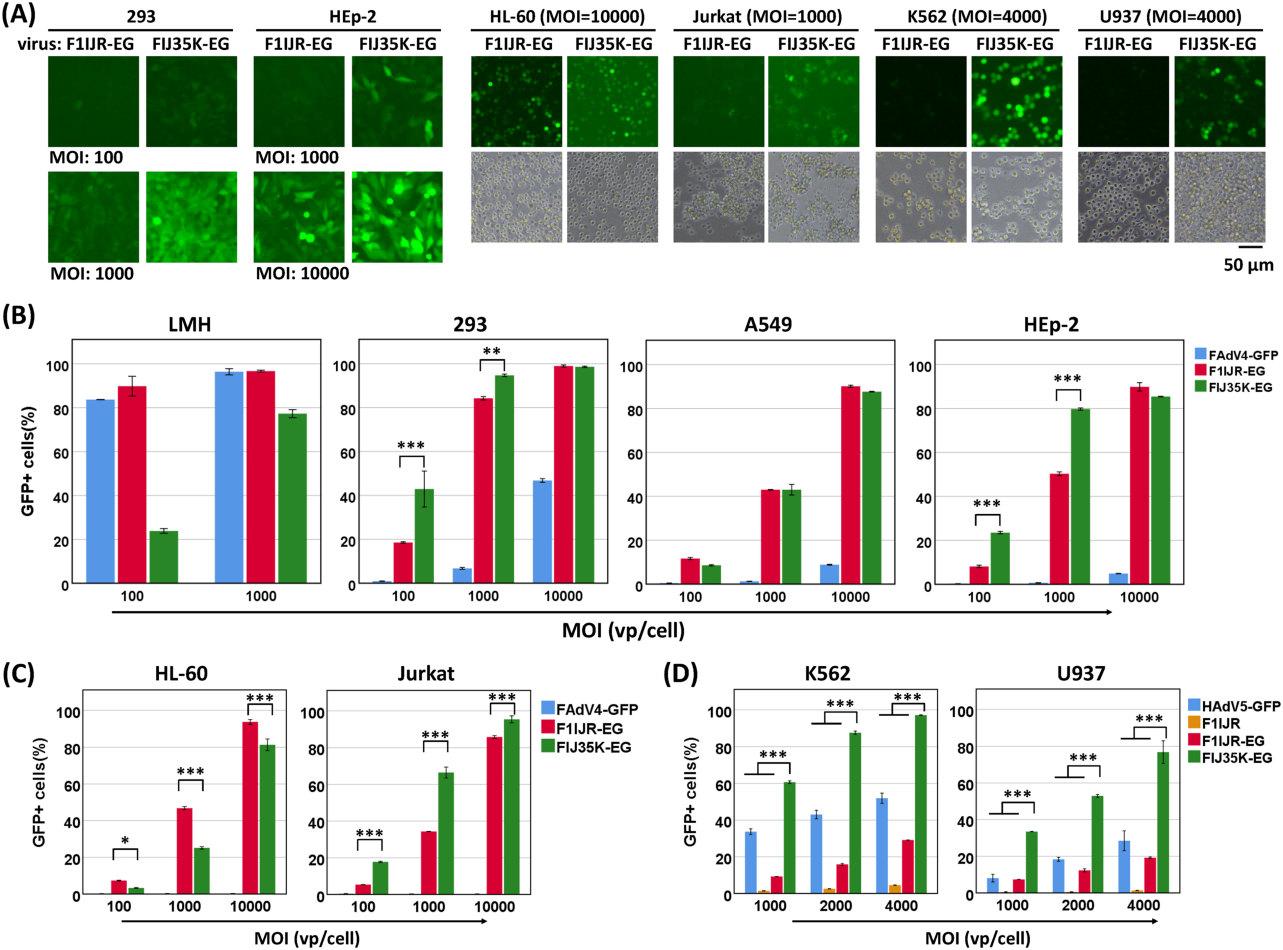
Fiber2 knob replacement with that of HAdV‐35 in FAdV‐4 vector enhanced gene transduction to human suspension cells. Adherent or suspension cells were infected with FAdV4F1IJR‐EG (F1IJR‐EG) or FAdV4FIJ35K‐EG (FIJ35K‐EG). F1IJR‐EG carried RGD4C insertion in the IJ loop of the fiber1 knob and the human EF1a promoter‐controlled GFP expression cassette. FAdV‐4 fiber2 knob in F1IJR‐EG was replaced with that of HAdV‐35 to generate FIJ35K‐EG. HAdV5‐GFP was an E1/E3‐deleted HAdV‐5 carrying the CMV promoter‐controlled GFP expression cassette. GFP expression was observed under a fluorescence microscope (A) before determined by flow cytometry 48 hours post infection. The results of flow cytometry for adherent cells are shown in (B), and those for suspension cells are shown in (C) and (D). Fiber knob replacement slightly improved GFP expression in adherent 293 and HEp‐2 cells but not in LMH or A549 cells; and a such modification significantly improved gene transduction in human suspension cells such as Jurkat, K562 and U937. All of the experiments were performed in duplicate and the data shown are from one representative experiment of the three experiments performed. *p < 0.05, **p < 0.01 and ***p < 0.001

Human EF1a promoter strongly drives constitutive expression in mammalian cells, which is especially suitable for expressing a transgene in suspension cells compared to the CMV promoter.^33^, ^40^, ^41^ Fiber knob substitution is a well‐known strategy for changing adenoviral tropism.^4^, ^28^, ^29^ HAdV‐35‐based vectors can efficiently transduce human hematopoietic cells because the virus receptor, CD46, is expressed abundantly on the membrane of these cells.^40^ Because fiber1 substitution makes FAdV‐4 defective in growth and fiber2 is a non‐essential gene,^31^ we replaced the knob of FAdV‐4 fiber2 with that of HAdV‐35 fiber in F1IJR. EF1a promoter moderately improved gene transfer to adherent cells: F1IJR‐EG transduced approximately 40% A549 or HEp‐2 cells at a MOI of 1000, which was two times as much as F1IJR did at the same MOI (Figures 3 and 4A, B). Fiber2 knob substitution further increased gene transduction to 293 and HEp‐2 cells, whereas it did not significantly affect A549 cells. It was worth noting that F1IJR‐EG or FIJ35K‐EG could transduce 85% of A549 or HEp‐2 cells at a MOI of 10,000, or 85% of 293 cells at a MOI of 1000 (Figure 4B), which suggested the modified viruses could transduce a large proportion of human adherent cells at an acceptable MOI.

The gene transfer efficiencies of F1IJR‐EG and FIJ35K‐EG were investigated with suspension human cells (Figure 4C, D; see also Supporting information, Figure S4). It was unexpected that both viruses could transduce HL‐60 cells with a high efficiency because previous work showed that HL‐60 was inert to the infection of HAdV‐5 or fiber‐pseudotyped HAdV‐5 vectors.^33^, ^42^ Both viruses could efficiently transduce Jurkat, K562 and U937 cells (Figure 4C; see also Supporting information, Figure S4). The efficiency of transduction to K562 or U937 cells was considerably high and the toxicity of FIJ35K‐EG caused cell lysis at a MOI of 10,000, which resulted in a decreasing percentage of GFP‐positive cells for K562 (see Supporting information, Figure S4). The gene transduction was further investigated with K562 and U937 cells within a restricted MOI range from 1000 to 4000 (Figure 4D). Although the transduction efficiency of F1IJR‐EG increased linearly as the MOIs increased, the highest efficiencies were still low (30% for K562 and 20% for U937). FIJ35K‐EG was obviously superior to F1IJR‐EG at all the tested MOIs. For K562 cells, the transduction of FIJ35K‐EG was 6.5, 5.5 and 3.3 times as high as that of F1IJR‐EG at the MOIs of 1000, 2000 and 4000, respectively. Similarly, FIJ35K‐EG was 4.6, 4.3 and 4.0 times more effective than F1IJR‐EG at various MOIs when transducing U937 cells. These data showed that the substitution to HAdV‐35 fiber knob significantly enhanced the transduction of FAdV‐4 vector to suspension cells.

### 3.5 Improved virus binding contributed to enhanced gene transduction

**FIGURE 5:**
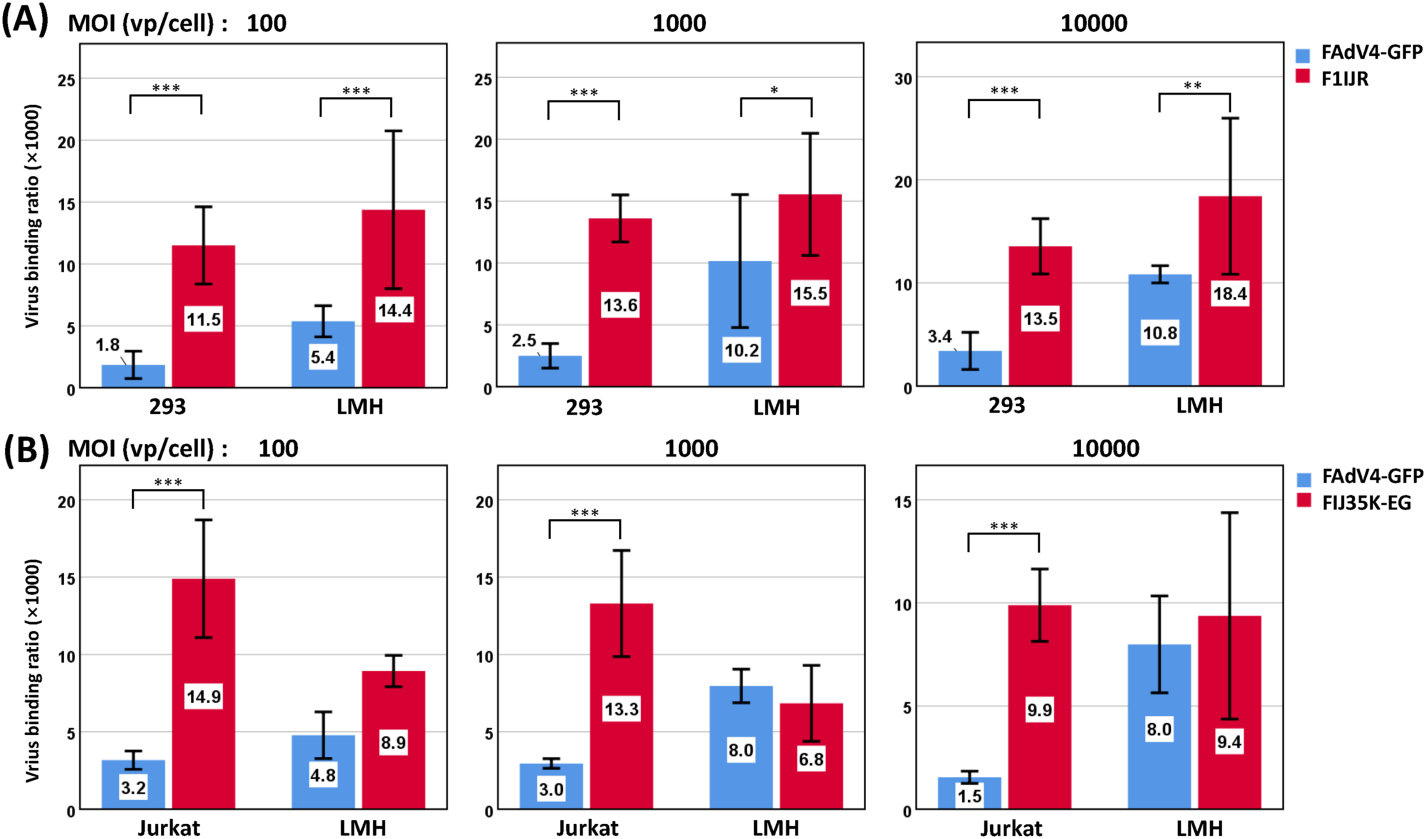
Virus binding of fiber‐modified FAdV‐4 to human cells. The viruses were allowed to bind to the cells for 2 hours at 4°C, and the amount of attached viruses was determined by real‐time PCR. (A) 293 cells, a representative of human adherent cells, were used as the target for the virus carrying RGD4C insertion (FAdV4F1IJR‐GFP) to bind. (B) Jurkat cells, a representative of human suspension cells, were used as the target for HAdV‐35 fiber pseudotyped FAdV‐4 (FAdV4FIJ35K‐EG) to bind. FAdV4‐GFP served as a control of unmodified FAdV‐4. The experiments were performed in triplicate and the data shown are from one representative experiment of the two performed. *p < 0.05, **p < 0.01 and ***p < 0.001

Fiber is the ligand of adenovirus for host cell attachment. Fiber modification aimed to promote gene transduction by strengthening the interaction between virus and target cell. We examined the binding of modified FAdV‐4 to target cells (Figure 5). Regarding the insertion of RGD4C to the IJ loop of the fiber1 knob, the binding of F1IJR to LMH or 293 cells increased compared to that of the parent virus FAdV4‐GFP. The virus binding ratios to 293 cells increased by 6.2, 5.5 and 4.0 times at MOIs of 100, 1000 and 10,000, respectively. The corresponding increases were 2.7, 1.5 and 1.7 times in the case of binding to LMH cells, suggesting that the insertion of RGD4C benefited gene transduction to 293 cells more significantly. For the knob replacement, the virus binding ratio of FIJ35K‐EG to Jurkat cells increased by 4.7, 4.5 and 6.4 times at MOIs of 100, 1000 and 10,000, respectively compared to that of FAdV4‐GFP. However, the difference of binding to LMH cells was not statistically significant between these viruses. These results suggested that fiber modification strengthened the interaction between recombinant FAdV‐4 and human cells, which finally led to enhanced gene transfer.

### 3.6 The growth of fiber‐modified FAdV‐4 in packaging cells

**FIGURE 6:**
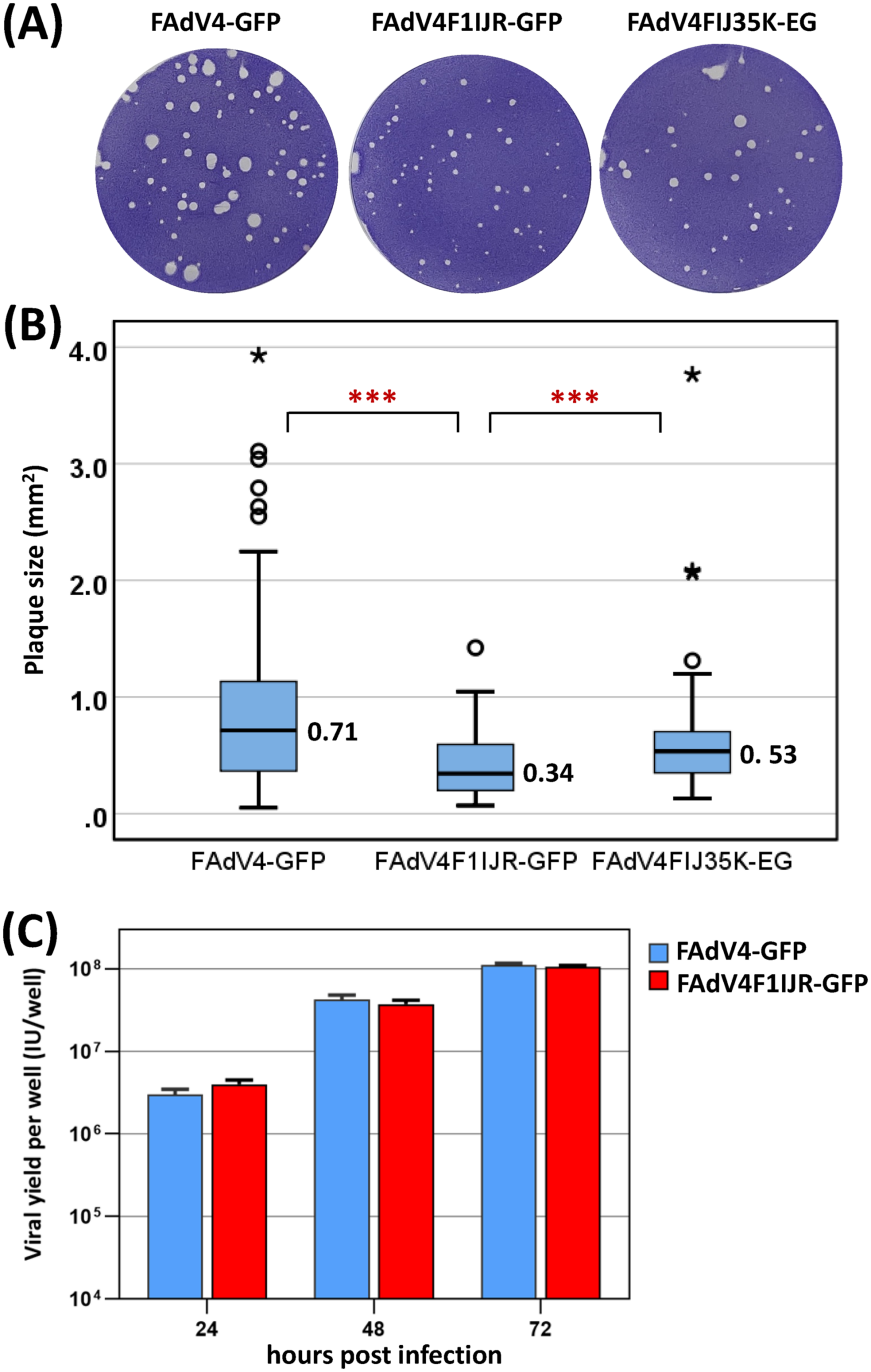
Growth of fiber‐modified FAdV‐4 vectors in packaging cells. (A) Plaque‐forming assay with chicken LMH cells. Seven days post infection, the plaques formed in semi‐solid culture medium were visualized by crystal violet staining. (B) Box plots of the plaque size. Plaques were photographed after crystal violet staining, and the area was measured using the Fiji image processing package (http://fiji.sc). (C) Virus replication in synchronously infected LMH cells. LMH cells in 12‐well plates were infected with FAdV4‐GFP or FAdV4F1IJR‐GFP at a MOI of 200 vp/cell for 2 hours. Progeny viruses were harvested at indicated time points post infection and titrated on LMH cells. The experiments were performed in duplicate and the data shown are from one representative experiment of the two performed. ***p < 0.001

The yield of progeny virus needs to be considered for the application of vectors. We first evaluated the plaque‐forming abilities of FAdV4‐GFP, F1IJR and FIJ35K‐EG on LMH cells (Figure 6A and B). FAdV4‐GFP formed the largest plaques, whereas F1IJR grew the smallest ones. Plaques originated from single infected LMH cells, and the growth of plaques depended on the virus replication cycle and the spreading of progeny virus. We further investigated the replication of FAdV4‐GFP and F1IJR when more than 90% packaging cells were infected synchronously. As shown in Figure 6C, after LMH cells were infected for 2 hours at a MOI of 200 vp/cell, the yields of progeny viruses were approximately the same for both viruses, suggesting a synchronous virus replication cycle. Collectively, it took longer for F1IJR to enrich to the indicated amount at the beginning, and F1IJR could produce progenies as efficiently as FAdV4‐GFP did in the subsequent rounds of amplification when a saturated infection was achieved.

## 4 DISCUSSION

Humans do not have pre‐existing immunity against FAdV, which has led to interest in constructing FAdV vectors for application in humans. CELO virus (FAdV‐A1) was constructed as a gene transfer tool two decades ago.^19^ HI loop modification of the fiber1 knob and polymer‐coating have been employed to improve the transduction of FAdV‐A1 to human cells.^22^, ^43^ Similar to FAdV‐A, FAdV‐C also has two types of fiber on the virion.^44^ It was reported recently that fiber1 was the major fiber for FAdV‐C4 adsorption.^45^, ^46^ We obtained the same findings and further confirmed that fiber2 was non‐essential for growing FAdV‐4 in a cultured chicken cell line,^31^ which implies that fiber1 may only be slightly modified, whereas fiber2 is free for radical reconstruction. Recently, chicken CAR homology was identified as a cellular receptor for FAdV‐4.^46^ However, FAdV‐4 vector could hardly transduce human cells. Here, to expand the application of FAdV‐4 vector, we mined the sites that could tolerate genetic modification in FAdV‐4 fibers.

Bioinformatics analysis was the first step for such a purpose. Although there is no structural information related to FAdV‐4 fibers, the crystal structures of FAdV‐1 fiber knobs have been resolved and made available.^20^, ^21^ According to the alignment of fiber knobs of FAdV‐1 and FAdV‐4,^38^ we selected four sites (i.e. the turning points where the peptide chain changes its direction) in different loops of the FAdV‐4 fiber1 knob. These sites in the CD‐, DE‐, HI‐ and IJ‐loops were also located on the surface of the fiber 1 knob domain. It was expected that insertion of exogenous peptide in these places would cause no steric hindrance to knob folding (Figure 2F). RGD4C was selected as the exogenous peptide in the present study.^23^, ^24^, ^27^ The FAdV‐4 viruses carrying RGD4C insertion could be all rescued and amplified in packaging cells, demonstrating that the selected sites were suitable for exogenous peptide integration, although the insertion in DE loop hampered the infection of the recombinant virus to LMH cells (Figure 3). Compared with unmodified FAdV4‐GFP, fiber modification significantly improved gene transfer to human adherent cell lines and the insertion to the IJ loop was the most effective (Figure 3). Considering the structural similarity of the FAdV knobs and the dispensable property of fiber2 in FAdV‐4, it was presumed that similar modifications could be conducted in the fiber2 knob. We inserted RGD4C into the CD loop of fiber2. The virus was successfully rescued and amplified, and efficiently transduced 293 or A549 cells, which confirmed the presumption (Figure 3A). These results illustrated that these four sites, especially the site in IJ loop, were the ideal positions in FAdV‐4 fibers for incorporation of exogenous peptide.

Besides short peptide insertion, knob substitution is another useful approach for changing the cellular tropism of adenoviruses.^4^, ^28^, ^29^ At the very beginning, the sequence encoding fiber1 shaft and knob of FAdV‐4 was replaced with that of HAdV‐35 in the laboratory. However, the recombinant virus could not be rescued in LMH cells, which led to the finding that fiber2, instead of fiber1, was dispensable for FAdV‐4 propagation.^31^ HAdV‐35 has a cellular receptor CD46, which is expressed abundantly on human suspension cells.^47^ The replacement of the fiber2 knob of FAdV‐4 with that of HAdV‐35 might enable a recombinant FAdV‐4 to transduce suspension cells. We examined the conception based on RGD4C insertion. The replacement of fiber2 knob impaired the infection of modified FAdV‐4 to chicken LMH cells (Figure 4B), and slightly improved the expression of reporter gene in 293 or HEp‐2 cells. As expected, such a modification significantly increased gene transfer to human suspension cells such as Jurkat, K562 and U937 (Figure 4C and D). These data suggested that RGD4C incorporation enhanced the infection of FAdV‐4 to human adherent cells, whereas HAdV‐35 fiber knob replacement worked more effectively for suspension cells.

We conducted virus binding experiments to explain the phenotype change of fiber‐modified FAdV‐4. RGD4C incorporation significantly improved virus binding to 293 cells and fiber knob replacement remarkably promoted virus attachment to Jurkat cells (Figure 5A and B), in agreement with the enhanced gene transfer. Interestingly, RGD4C incorporation slightly increased virus binding to LMH cells (Figure 5A), whereas it did not result in improved virus infection (Figure 3B, LMH infected at a MOI of 100). The fiber knob substitution did not significantly affect the binding (Figure 5B), whereas it partially impaired the infection to LMH cells (Figure 4B). These phenomena implied that other steps besides virus binding, such as cell entry, escape from endosome or transport of viral genome to the nucleus, might influence FAdV‐4 infection in these situations.^48^

The production of recombinant virus is crucial for the use of viral vector. FAdV cannot replicate in human cells because of the host species barrier between birds and mammals.^2^, ^49^ To our knowledge, up to now, all of the developed FAdV vectors are replication‐competent and can be amplified in LMH or other chicken cell lines. A plaque‐forming assay could provide virus growth and spreading information in the case of only a few cells in a monolayer culture system being infected by single virions. Fiber modification led to the change of plaque size (Figure 6A and B), which might result from the alteration of virus binding, in the case of F1IJR, or from combined changes in other stages of virus replication, in the case of FIJ35K‐EG with the deletion of the FAdV‐4 ORF19A gene. When a high MOI was applied and more than 90% packaging cells were synchronously infected in the first replication cycle, seed viruses such as F1IJR, which formed smaller plaques, did not certainly produce fewer progeny viruses. The virus yields were very close between F1IJR and the control virus FAdV4‐GFP (Figure 6C).

Fiber modification substantially improved the gene transduction of FAdV‐4 vectors to human adherent or suspension cells. However, compared to human adenoviruses, modified FAdV‐4 could only transduce human cells with a low efficiency (see Supporting information, Figure S5). Human adenoviruses often need a co‐receptor besides the main receptor of fiber for virus entry. The interaction between fiber and the main receptor helps the virus attach and bind to the cellular surface, followed by the interaction between the viral penton base and the co‐receptor, which occurs in a short distance and facilitates virus endocytosis.^47^, ^48^ For human adenoviruses, it is the RGD motif in the penton base that interacts with the co‐receptor of integrins on the cellular membrane. FAdVs do not have a RGD motif in the penton base, and their entry to host cells remains to be fully elucidated. It is an interesting question and deserves further research regarding whether including a RGD motif in the FAdV‐4 penton base can promote gene transduction to human cells.

In conclusion, the present study has identified four sites in the FAdV‐4 fiber1 knob for the insertion of exogenous peptide and has demonstrated that the fiber2 knob was replaceable. In addition, RGD4C insertion to the fiber1 knob and the replacement of fiber2 knob with that of HAdV‐35 enabled FAdV‐4 vectors to transduce human cells. These findings could be helpful for the application of FAdV‐4 vectors in human gene therapy and vaccine development.

## CONFLICT OF INTEREST

A patent application has been filed on this work by National Institute for Viral Disease Control and Prevention, Chinese Center for Disease Control and Prevention.

## AUTHOR CONTRIBUTIONS

ZL was responsible for the study conceptualization. ZL was responsible for funding acquisition. WZ, XG, FY and WH were responsible for the investigation. XZ and ZL were responsible for the methodology. XG and XZ were responsible for project administration. ZL and XZ were responsible for resources. ZL was responsible for study supervision. WZ and XG were responsible for study validation. ZL was responsible for writing the original draft, as well as reviewing and editing.

## Supporting information


**Figure S1.** Schematic diagram of the construction of the shuttle plasmid pMD‐FAV4F1CDR, which carried the insertion of RGD4C in the CD loop of the FAdV‐4 fiber1 knob.
**Figure S2**. Schematic diagram of the construction of the adenoviral plasmid pKFAV4F1CDR‐GFP using the method termed restriction‐assembly^1,2^.
**Figure S3.** Schematic diagram of the construction of the adenoviral plasmid pKFAV4F2CDR‐GFP, in which the RGD4C coding sequence was inserted into the CD loop of the FAdV‐4 fiber2 knob.
**Figure S4.** Transduction of suspension K562 and U937 cells with fiber2 pseudotyped FAdV‐4 vector. Cells were infected with FAdV4F1IJR‐EG (F1IJR‐EG), FAdV4FIJ35K‐EG (FIJ35K‐EG) or FAdV4‐GFP (control). F1IJR‐EG carried the RGD4C insertion in the IJ loop of the fiber1 knob and the EF1a promoter‐controlled GFP expression cassette. The Fiber2 knob in F1IJR‐EG was replaced with that of HAdV‐35 to generate FIJ35K‐EG. GFP expression was determined with flow cytometry 48 hours post infection. When FIJ35K‐EG was used at a high MOI of 10,000 vp/cell, efficient transduction caused cell lysis, which led to decreased GFP fluorescence. All of the experiments were performed in duplicate and the data shown are from one representative experiment of the two that were performed.
**Figure S5.** Comparisons of gene transduction between human adenovirus 5 (HAdV‐5) and fiber‐modified fowl adenovirus 4 (FAdV‐4) vectors. HAdV5‐GFP was the E1/E3‐deleted HAdV‐5 carrying cytomegalovirus (CMV) promoter‐controlled green fluorescent protein (GFP) expression cassette in the original E1 region, and the CMV promoter in HAdV5‐GFP was replaced with that of human EF1a to generate HAdV5‐EG. Chicken LMH cells, as well as human 293, A549 and HEp‐2 cells, were infected with HAdV5‐GFP, HAdV5‐EG or FAdV4F1IJR‐GFP at various MOIs (vp/cell) for 4 hours. The percentages of GFP+ cells were determined by flow cytometry at 48 hours post infection. The data shown are from one representative experiment.

## Data Availability

The data that support the findings of this study are available from the corresponding author upon reasonable request.
