# Supplementary material for: Fiber modifications enable fowl adenovirus 4 vectors to transduce human cells
Source: J Gene Med. 2021 Jun 11;23(10):e3368. doi: 10.1002/jgm.3368 (PMC8518954; doi:10.1002/jgm.3368)
Supplement: Supplementary file 1 — Figure S1. Schematic diagram of the construction of the shuttle plasmid pMD‐FAV4F1CDR, which carried the insertion of RGD4C in the CD loop of the FAdV‐4 fiber1 knob. Figure S2. Schematic diagram of the construction of the adenoviral plasmid pKFAV4F1CDR‐GFP using the method termed restriction‐assembly1,2. Figure S3. Schematic diagram of the construction of the adenoviral plasmid pKFAV4F2CDR‐GFP, in which the RGD4C coding sequence was inserted into the CD loop of the FAdV‐4 fiber2 knob. Figure S4. Transduction of suspension K562 and U937 cells with fiber2 pseudotyped FAdV‐4 vector. Cells were infected with FAdV4F1IJR‐EG (F1IJR‐EG), FAdV4FIJ35K‐EG (FIJ35K‐EG) or FAdV4‐GFP (control). F1IJR‐EG carried the RGD4C insertion in the IJ loop of the fiber1 knob and the EF1a promoter‐controlled GFP expression cassette. The Fiber2 knob in F1IJR‐EG was replaced with that of HAdV‐35 to generate FIJ35K‐EG. GFP expression was determined with flow cytometry 48 hours post infection. When FIJ35K‐EG was used at a high MOI of 10,000 vp/cell, efficient transduction caused cell lysis, which led to decreased GFP fluorescence. All of the experiments were performed in duplicate and the data shown are from one representative experiment of the two that were performed. Figure S5. Comparisons of gene transduction between human adenovirus 5 (HAdV‐5) and fiber‐modified fowl adenovirus 4 (FAdV‐4) vectors. HAdV5‐GFP was the E1/E3‐deleted HAdV‐5 carrying cytomegalovirus (CMV) promoter‐controlled green fluorescent protein (GFP) expression cassette in the original E1 region, and the CMV promoter in HAdV5‐GFP was replaced with that of human EF1a to generate HAdV5‐EG. Chicken LMH cells, as well as human 293, A549 and HEp‐2 cells, were infected with HAdV5‐GFP, HAdV5‐EG or FAdV4F1IJR‐GFP at various MOIs (vp/cell) for 4 hours. The percentages of GFP+ cells were determined by flow cytometry at 48 hours post infection. The data shown are from one representative experiment. [file JGM-23-e3368-s001.docx]

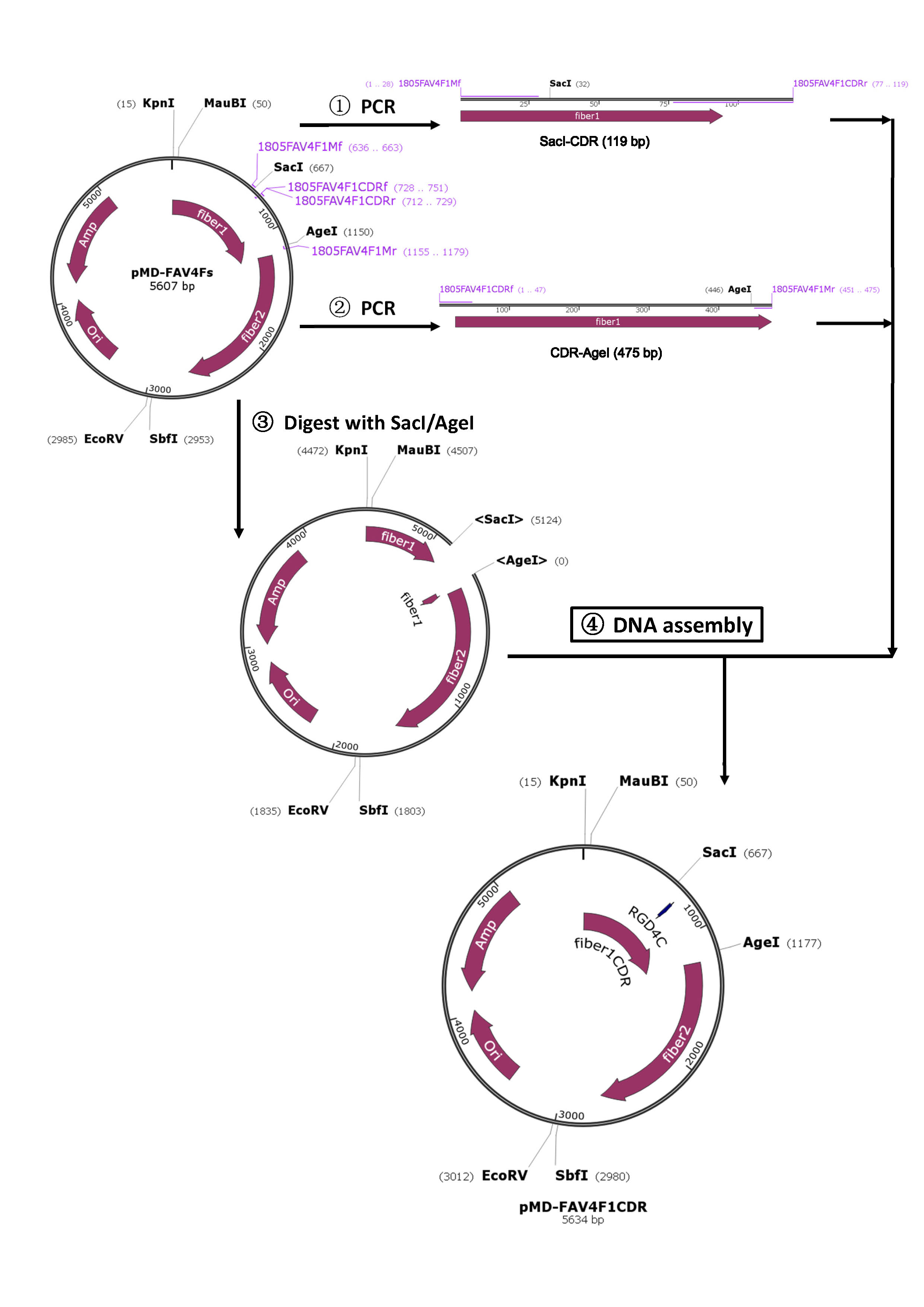


**Figure S1.** Schematic diagram of constructing shuttle plasmid pMD-FAV4F1CDR, which carried the insertion of RGD4C in the CD loop of FAdV-4 fiber1 knob.


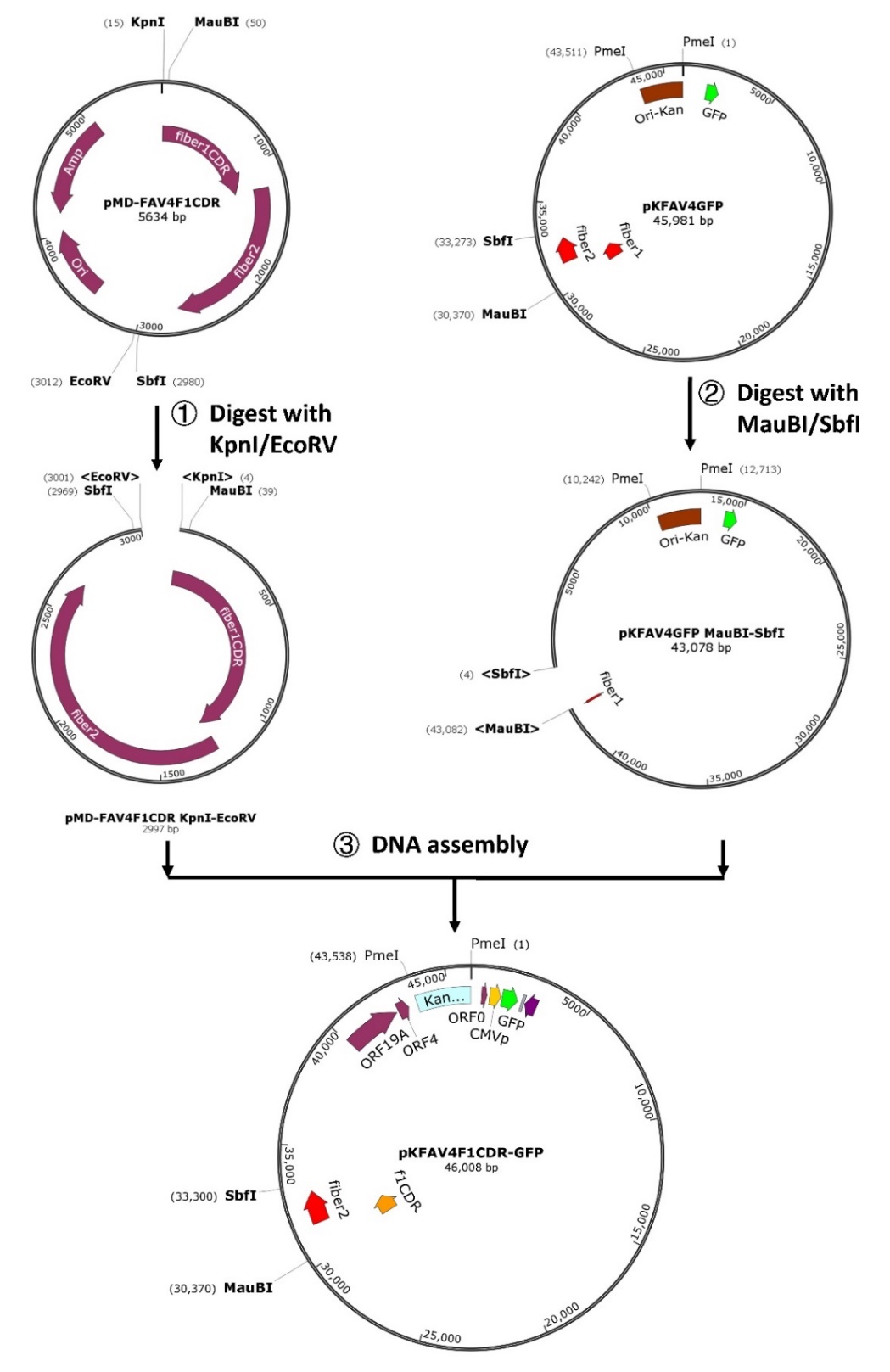


**Figure S2**. Schematic diagram of constructing adenoviral plasmid pKFAV4F1CDR-GFP with the method we called restriction-assembly ^1,2^.

**Reference**

1. Guo X, Mei L, Yan B, Zou X, Hung T, Lu Z. Site-directed modification of adenoviral vector with combined DNA assembly and restriction-ligation cloning. *J Biotechnol.* 2020;307:193-201.

2. Liu H, Lu Z, Zhang X, et al. Single Plasmid-Based, Upgradable, and Backward-Compatible Adenoviral Vector Systems. *Hum Gene Ther.* 2019;30(6):777-791.


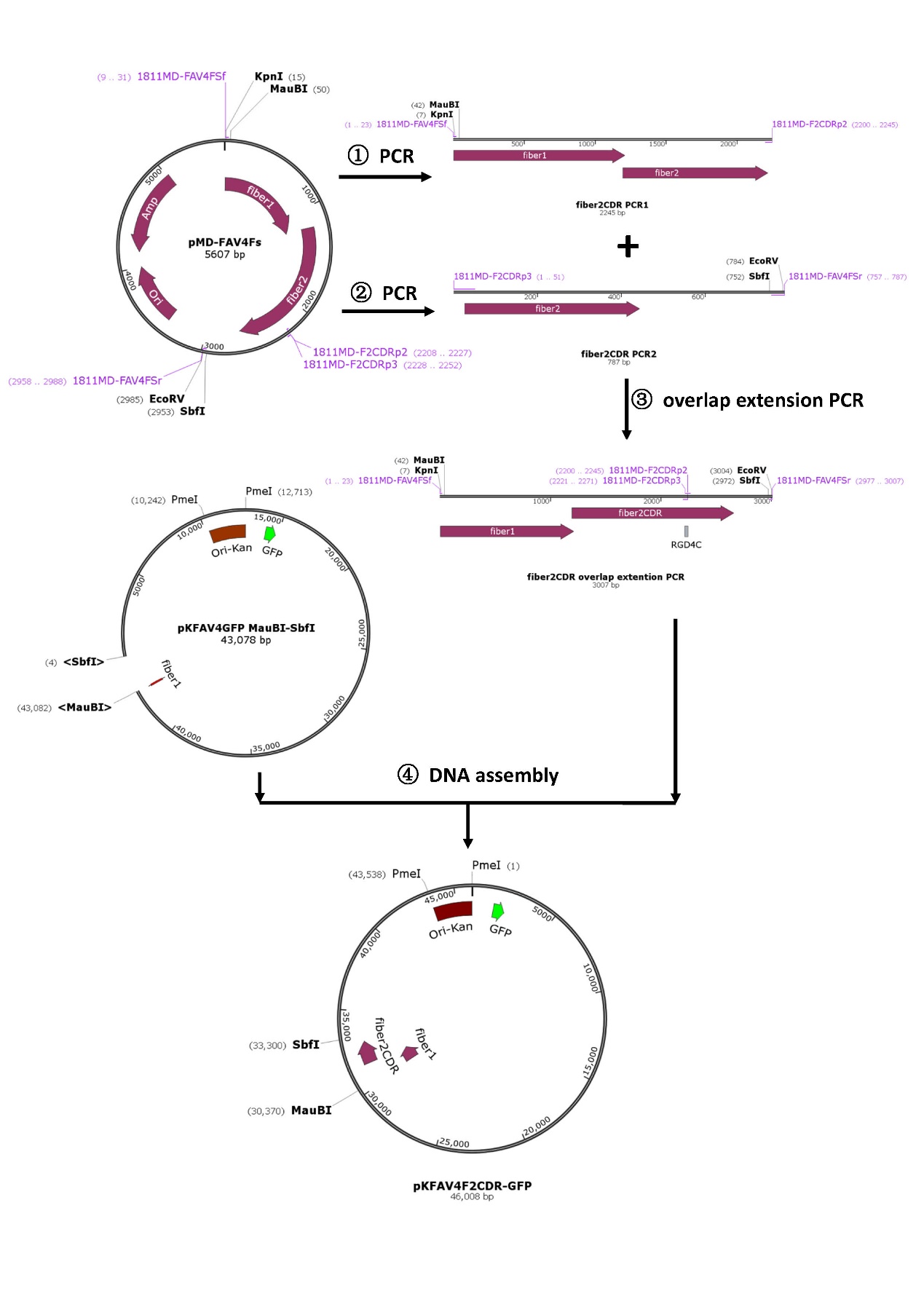


**Figure S3.** Schematic diagram of constructing adenoviral plasmid pKFAV4F2CDR-GFP, in which RGD4C coding sequence was inserted into the CD loop of FAdV-4 fiber2 knob.


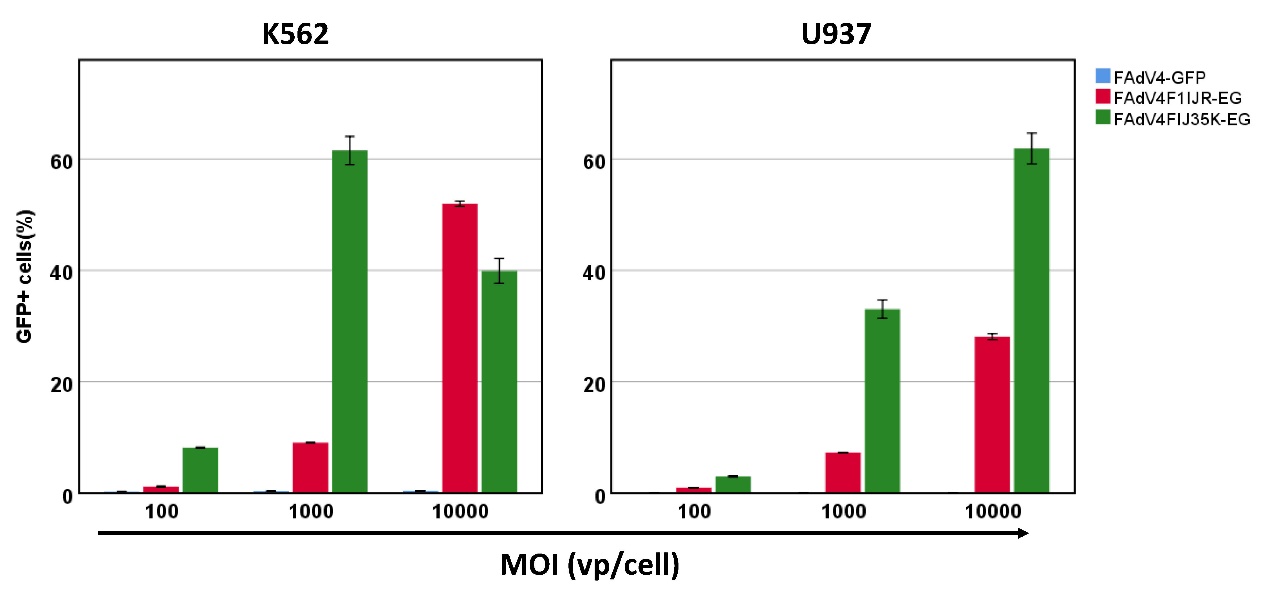


**Figure S4.** Transduction of suspension K562 and U937 cells with fiber2 pseudotyped FAdV-4 vector. Cells were infected with FAdV4F1IJR-EG (F1IJR-EG), FAdV4FIJ35K-EG (FIJ35K-EG) or FAdV4-GFP (control). F1IJR-EG carried RGD4C insertion in IJ loop of fiber1 knob and EF1a promoter-controlled GFP expression cassette. Fiber2 knob in F1IJR-EG was replaced with that of HAdV-35 to generate FIJ35K-EG. GFP expression was determined with flow cytometry 48 hours post infection. When FIJ35K-EG was used at a high MOI of 10000 vp/cell, efficient transduction caused cell lysis, which led to the falling of GFP fluorescence. All the experiments were performed in duplicate and the data shown were from one representative experiment of the two performed.


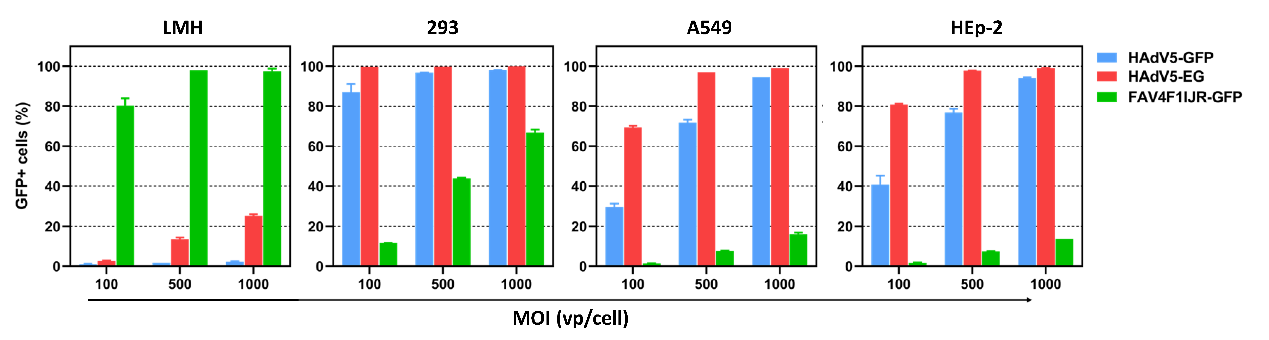


**Figure S5.** Comparisons of gene transduction between human adenovirus 5 (HAdV-5) and fiber-modified fowl adenovirus 4 (FAdV-4) vectors. HAdV5-GFP was the E1/E3-deleted HAdV-5 carrying CMV promoter-controlled GFP expression cassette in original E1 region, and the CMV promoter in HAdV5-GFP was replaced with that of human EF1a to generate HAdV5-EG. Chicken LMH cells as well as human 293, A549 and HEp-2 cells were infected with HAdV5-GFP, HAdV5-EG or FAdV4F1IJR-GFP at various MOIs (vp/cell) for 4 hours. The percentages of GFP+ cells were determined by flow cytometry at 48 hours post infection. The data shown were from one representative experiment.
